# Endogenous Synthesis of n-3 Polyunsaturated Fatty Acids in Fat-1 Mice Is Associated with Increased Mammary Gland and Liver Syndecan-1

**DOI:** 10.1371/journal.pone.0020502

**Published:** 2011-05-31

**Authors:** Haiguo Sun, Yunping Hu, Zhennan Gu, Martha D. Wilson, Yong Q. Chen, Lawrence L. Rudel, Mark C. Willingham, Iris J. Edwards

**Affiliations:** 1 Department of Pathology, Wake Forest School of Medicine, Winston-Salem, North Carolina, United States of America; 2 Department of Cancer Biology, Wake Forest School of Medicine, Winston-Salem, North Carolina, United States of America; Universidade de São Paulo, Brazil

## Abstract

Long chain n-3 PUFA have been shown to have chemopreventive properties against breast cancer through various mechanisms. One pathway, studied in human breast cancer cell lines, involves upregulation of the proteoglycan, syndecan-1 (SDC-1) by n-3 PUFA-enriched LDL. Using Fat-1 mice that are able to convert n-6 to n-3 PUFA, we tested whether SDC-1 level in vivo is elevated in mammary glands due to endogenously synthesized rather than LDL-derived n-3 PUFA. Female Fat-1 and wild type (wt) mice were fed an n-6 PUFA- enriched diet for 7 weeks. Fatty acid analysis of plasma lipoproteins showed that total n-6 PUFA reflected dietary intake similarly in both genotypes (VLDL, 36.2±2.2 and 40.9±3.9; LDL, 49.0±3.3 and 48.1±2.0; HDL, 54.6±1.2 and 58.2±1.3, mean ± SEM percent of total fatty acids for Fat-1 and wt animals respectively). Lipoprotein percent n-3 PUFA was also similar between groups. However, phospholipids and triglycerides extracted from mammary and liver tissues demonstrated significantly higher n-3 PUFA and a corresponding decrease in the ratio n-6/n-3 PUFA in Fat-1 compared to wt mice. This was accompanied by higher SDC-1 in mammary glands and livers of Fat-1 mice, thus demonstrating that endogenously synthesized n-3 PUFA may upregulate SDC-1 in the presence of high dietary n-6 PUFA.

## Introduction

A large body of evidence now points to protective role against breast cancer for the long chain marine n-3 polyunsaturated fatty acids (PUFA), eicosapentaenoic acid (EPA, 20:5n-3) and docosahexaenoic acid (DHA, 22:6n-3). Due to a lack of the necessary desaturases, these essential fatty acids cannot be synthesized de novo by mammals and must be obtained from diet. Human population studies have demonstrated an inverse relationship between breast cancer incidence and calories from fish oil [1], [2]. In addition, data from 20 countries not only identified a negative relationship with fish oil consumption, but also a positive correlation between breast cancer and intake of saturated and n-6 polyunsaturated fat [3]. Animal studies have provided strong support for a role of dietary fats in breast cancer. In chemical carcinogen-induced breast cancer in rats [4], [5], [6], [7], [8] and human cancer cell xenografts in nude mice [9], [10], [11], tumor growth rate, size and metastases were all suppressed by n-3 PUFA and promoted by n-6 PUFA diets. In more recent studies with HER-2/neu transgenic mouse models of spontaneous breast cancer, a fish oil diet compared to a corn oil diet increased the latency time to tumor development, reduced the number of tumors and was associated with a lower grade of mammary gland histopathology [12], [13].

As reviewed [14], [15], insights into the mechanisms responsible for the anti-breast cancer properties of n-3 PUFA have been provided largely by in vitro investigations with human breast cancer cell lines. Of several mechanisms proposed, the most frequently cited for their anti-cancer activity is the ability of n-3 PUFA to block the metabolism of the n-6 PUFA, arachidonic acid (AA) and linoleic acid (LA) into compounds that promote the malignant phenotype of cancer cells [14], [15], [16], [17], [18], [19]. This may involve differential activation of the nuclear receptor, PPARγ and/or differential regulation of intracellular signaling pathways by n-3 and n-6 PUFA. Our previous in vitro studies have defined a novel pathway whereby n-3 PUFA-enriched LDL inhibits human breast cancer cell growth: the n-3 PUFA, DHA activates PPARγ which results in transcriptional up-regulation of the *Sdc-1* target gene and the syndecan-1 (SDC-1) protein induces apoptosis [20], [21], [22]. SDC-1 is the primary cell surface proteoglycan of epithelial cells and has been implicated in a number of regulatory processes in tumorigenesis including adhesion [23], [24], [25], [26], sequestration and storage of growth factors for which it may serve as a co-receptor [27], [28], [29], invasion [30], [31] and induction of apoptosis [22], [32]. Its in vivo regulation by n-3 PUFA may therefore have a profound effect on breast cancer growth and progression.

The Fat-1 mouse was engineered by Kang et al [33] to express the *fat-1* gene from *Caenorhabditis elegans*. This encodes an n-3 desaturase that can convert n-6 to n-3 fatty acids to result in increased levels of n-3 PUFA in all major tissues. Previous reports have not addressed effects of the n-3 PUFA enrichment on genes that are regulated by these fatty acids. The use of this model allowed us to determine that SDC-1 can be upregulated in vivo by tissue n-3 PUFA in the absence of n-3 PUFA-enriched lipoproteins.

## Materials and Methods

### Mice and diets

Animal care was conducted in compliance with the state and federal Animal Welfare Acts and standards and policies of the Department of Health and Human Services. The protocol (A09–131) was approved by the Wake Forest University Animal Care and Use Committee. Fat-1 transgenic mice were generously supplied by Dr. Jing X. Kang, Department of Medicine, Massachusetts General Hospital and Harvard Medical School, Boston, Massachusetts. The genetic groups (Fat-1 and wild type littermates) consisted of 5 female animals. All animals were housed in an isolated environment in barrier cages and a fed specific diet for 7 weeks after weaning. Diets were prepared by the custom animal diet laboratory of the Animal Resources Program at Wake Forest University and contained 397 kcal/100g; 30% of energy was from fat, 50% from carbohydrates and 30% from proteins. The fatty acid content was 30% saturated (16∶0+18∶0), 26% monounsaturated (18∶1) and 43% n-6 polyunsaturated (18∶2). Detailed diet composition has been previously described [34]. At termination, mice were fasted for 4 h and then euthanized. A blood sample was immediately drawn via heart puncture.

### Plasma lipids and lipoproteins

The terminal blood samples were placed into tubes containing a protease inhibitor cocktail (Sigma) dissolved in 5% EDTA, 5% NaN_3_. Plasma was separated by centrifugation at 12,500 g for 15 min, 4°C. Lipoproteins (VLDL, LDL, and HDL) were isolated by gel filtration chromatography of whole plasma injected onto a Superose 6 10/300 GL column (GE Healthcare) and lipoproteins eluted with 0.9% saline, 0.01% EDTA and 0.01% NaN_3_ at 0.4 ml/min. The HPLC chromatography system used was a LaChrom Elite (Hitachi High Technologies) consisting of an L-2200 Autosampler with Peltier cooling, an L-2420 UV-VIS Detector, and an L-2100 SMASH pump. Fatty acid distributions in whole plasma and isolated lipoproteins were determined by gas chromatography following methylation of the fatty acid moieties. Fatty acid methyl esters were separated and quantified by gas chromatography (GC) on a CP Select for FAME capillary column (100 m×0.25 mm id, Varian) installed in an Agilent 6890N gas chromatograph with a programmable cool on-column capillary inlet, flame ionization detector (FID), and Agilent 7683B autosampler/injector. Chromatographic data collection and analysis was via Chrom Perfect™ Spirit Chromatography Data System (version 5.0) in Microsoft Windows XP Pro. The chromatographic conditions were: H_2_ carrier gas, 20 psi head pressure, 1.25 ml/min at 90°C; N_2_ make-up gas, 23 ml/min; inlet temperature at 3°C above the oven temperature; and FID at 230°C. The oven temperature was programmed to begin at 90°C and hold for 0.5 min, increase at 10°C per min to 150°C, increase at 2.5°C per min to 200°C, increase at 1.5°C per min to the final temperature, 220°C, and hold at 220°C for 20 min.

### Mammary gland and liver lipid analysis

At necropsy, mammary glands and livers were dissected, washed twice with balanced salt solution (BSS [137 mM NaCl, 2.7 mM KCl, 1.45 mM KH_2_PO_4_, 20.3 mM Na_2_HPO_4_, pH 7.4]), and divided into sections for lipid, protein, mRNA and immunohistochemical analyses. Lipids were extracted with chloroform-methanol and lipid classes were separated by thin layer chromatography. Bands corresponding to triglyceride (TG) and phospholipid (PL) were scraped and methyl esters were made by addition of NaOH in methanol, followed by boron trifluoride in methanol. Fatty acids were quantified by gas chromatography as above.

### Determination of cholesterol, triglyceride and phospholipid content of tissues

Aliquots of chloroform extracts were evaporated to dryness under nitrogen, and exchanged into aqueous solution by addition of 2 ml of 1% Triton X -100 in chloroform, re-evaporation and solubilization of the detergent residue in 0.5 ml dH_2_O. Total cholesterol (TC) and TG were measured using standard enzymatic procedures [35]. PL was measured in a separate aliquot as inorganic phosphorous [36].

### Western blot assay

Mouse mammary and liver tissues were homogenized and lysed in ice-cold lysis buffer (25 mM Tris-HCl, 150 mM NaCl, 1% Triton X-100, 0.1 mg/ml PMSF) with 1× Proteinase and 1×Phosphatase inhibitors (Roche Applied Science). For the analysis of SDC-1 protein, lysates were dialyzed against heparitinase buffer (50 mM HEPES, 50 mM NaOAc, 150 mM NaCl, 5 mM CaCl_2_) for 24 h at 4°C and then digested by chondroitinase ABC (Seikagaku, America) and heparinase III (Sigma-Aldrich Company, Allentown, PA) at 37°C overnight. Protein extracts were prepared for Western blot analysis as described earlier using indicated antibodies [37]. Band densities on photographic films were analyzed using Image J 1.37v (NIH).

### Quantification of mRNA levels

Total mRNA was extracted from mammary glands and liver of five wild type and Fat-1 mice with RNeasy® Protect Mini Kit (Cat. No. 74124, QIAGEN, Maryland). Quantitative real-time PCR (RT-PCR) was performed as described [21], [22]. Amplification reactions were performed in triplicate in Applied Biosystems 7500 Real-Time PCR System. The primers used for mouse SDC-1 were 5′-tggagaacaagacttcacctttg-3′ (forward) and 5′-ctcccagcacttccttcct-3′ (reverse), mouse SDC-4 were 5′-atggacctggagccagctcacccccaa-3′ (forward) and 5′-tcatgcgtagaactcattggtggg-3′ (reverse), mouse perlecan were 5′-caagcagtttctgggcaacaaggt-3′ (forward) and 5′-acccacgaggatcacatctggttt-3′ (reverse), mouse versican were 5′-tggatcatctggatggcgatgtgt-3′ (forward) and 5′-caaagccatttctccaagctgcct-3′ (reverse), mouse decorin were 5′-acctctcgtgaagttggaaaggct-3′ (forward) and 5′-tccgcagcttggtgatctcattct-3′ (reverse), and mouse biglycan were 5′-caacaacatcaccaaggtgggcat-3′ (forward) and 5′-tggcaaccactgcctctacttctt-3′ (reverse). Primers for mouse peptidylprolylisomerase B (PPIB) housekeeping gene were 5′-gcccaaagtcaccgtcaa (forward) and 5′-tccgaagagaccaaagatcac (reverse). Care was taken to design the primers so as to minimize amplification from any contaminating genomic DNA. Melt curve analyses performed at the end of the real-time PCR reproducibly show a single peak. SDC-1, SDC-4, perlecan, versican, decorin and biglycan data were normalized to the housekeeping control PPIB.

### Immunohistochemical Analysis

Mammary tissues were fixed in buffered formalin at 4°C, embedded in paraffin, sectioned and deparaffinized. Immunostaining was conducted using a rabbit polyclonal antibody raised against a recombinant protein corresponding to amino acids 82-256 of human SDC-1 (H174 from Santa Cruz Biotechnology, Santa Cruz, CA) and the DAKO EnVion +system peroxidase 3, 3′-diaminobenzidine (DAB) kit according to the manufacturer's instructions.

### Statistical analyses

Data are expressed as mean ± SD or SEM. Results were analyzed by Student's *t* test and differences were considered significant at *P*<0.05.

## Results

### Plasma lipoproteins of Fat-1 mice fed an n-6 PUFA diet are deficient in n-3 PUFA

**Figure 1:**
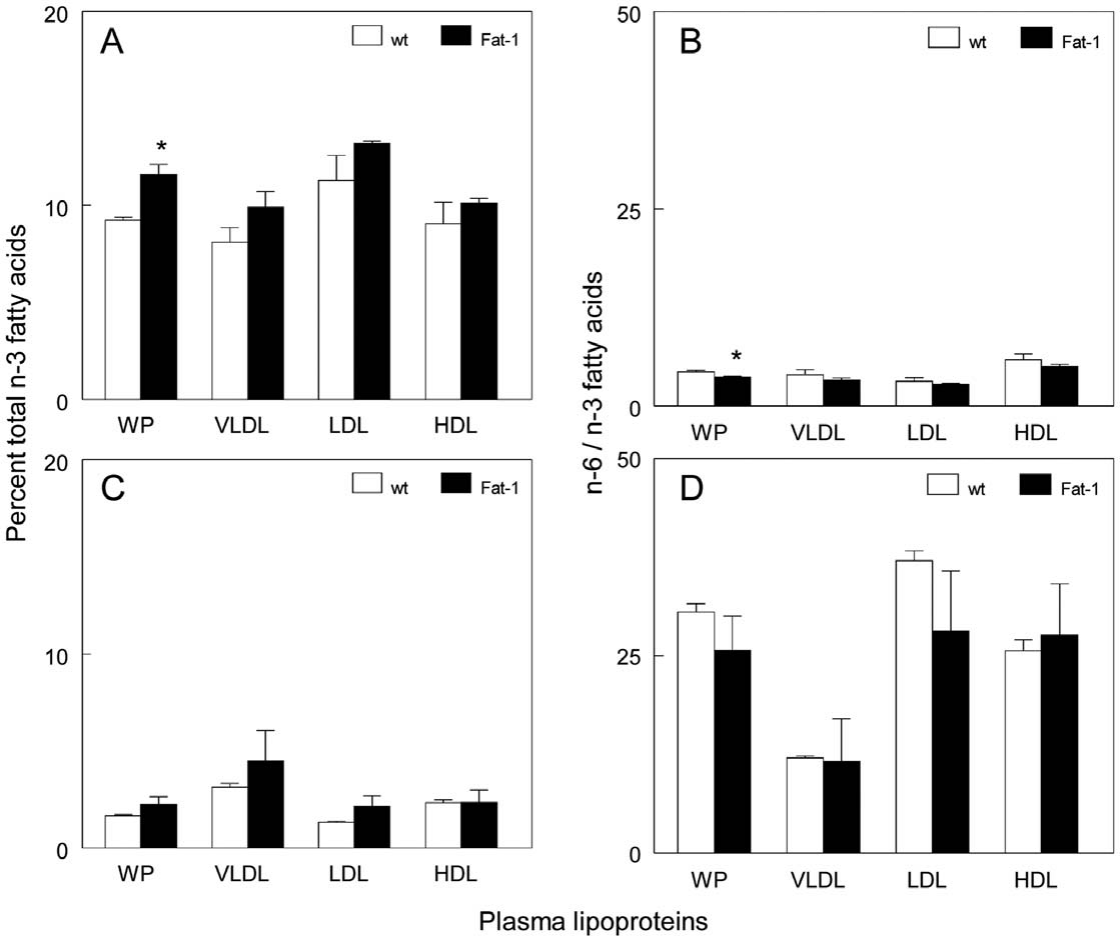
Fatty acid composition of whole plasma (WP) and lipoproteins of Fat-1 and wild type (wt) mice fed a chow control (A, B) and n-6 PUFA-enriched diet (C, D). Lipoproteins were isolated by gel filtration chromatography and fatty acids were measured as methyl esters by gas chromatography. Bars represent the means ± SEM of A and C, total n-3 PUFA (eicosapentaenoic acid, 20∶5; docosapentaenoic acid, 22∶5; docosahexaenoic acid, 22∶6); B and D, ratio of n-6 PUFA (linoleic acid, 18∶2+ arachidonic acid, 20∶4) to n-3 PUFA . * *P*<0.05 Fat-1 versus wt by Student's *t* test.

The effects of the n-6 PUFA enriched diet on plasma lipoproteins are shown in Fig. 1. When Fat-1 and wt mice were fed a commercial chow control diet (n-6 to n-3 ratio, approximately 5-8 to 1) significantly higher n-3 PUFA were measured in the whole plasma of Fat-1 compared to wt mice (Fig. 1A). This was presumably due in part to elevated n-3 PUFA free fatty acids because percent n-3 PUFA in isolated lipoproteins was similar between Fat-1 and wt mice. In agreement with these data, n-6/n-3 ratios in Fat-1 compared to wt mice were significantly lower in whole plasma only and not in isolated lipoproteins (Fig. 1B). Seven weeks of the n-6 PUFA diet (n-6 to n-3 ratio of 40) resulted in an elevation in plasma n-6 PUFA with a corresponding decrease in n-3 PUFA for both genotypes (Fig. 1C versus 1A). A similar reduction in percent n-3 PUFA for Fat-1 and wt mice was observed for all lipoprotein species. This resulted in a marked increase in the n-6/n-3 PUFA ratios of whole plasma and isolated lipoproteins with no differences in response between the genotypes (Fig. 1D versus 1B). Thus the lipoproteins directly reflected dietary PUFA intake with little influence of the *fat-1* transgene.

#### Mammary tissue and liver lipids of Fat-1 mice fed an n-6 PUFA diet are enriched in n-3 PUFA

No differences were observed in the percent distribution of mammary tissue lipids which was 98.7±0.1 and 98.9±0.2 in TG, 1.0±0.1 and 0.8±0.4 in PL, 0.3±0.03 and 0.2±0.08 in TC, mean ± SEM for Fat-1 and wt respectively. These lipid classes were separated by TLC and analyzed for fatty acid content. The percent fatty acid composition of mammary tissue PL is shown in Table 1 and of TG in Table 2. For both of these lipids, there was no difference between Fat-1 and wt mice for total saturated and monounsaturated fatty acids. Levels of n-3 PUFA, EPA, (20∶5) and docosapentaenoic acid (DPAn-3 , 22∶5), were significantly higher in PL of Fat-1 compared to wt mice and this resulted in a 44% lower n-6/n-3 ratio in Fat-1 relative to wt (Table 1). The n-6/n-3 ratio was likewise significantly lower in the TG fraction from Fat-1 relative to wt mice (Table 2). In TG however, the higher n-3 PUFA in Fat-1 mice was due to an increase in DHA (22∶6), as well as in DPAn-3.

**Table 1:** Mouse mammary gland phospholipid fatty acid composition.

Fatty acid	Wild type	Fat-1
14∶0	0.8±0.1	0.5±0.4^a^
16∶0	21.9±1.5	21.9±1.2
16:1n-7	2.0±0.2	1.8±0.4
18∶0	20.5±1.0	19.8±0.9
18:1n-9	14.5±1.0	15.8±3.1
18:2n-6	15.9±1.4	14.4±2.5
18:3n-3	0.1^b^	0.1^c^
20:3n-6	0.8±0.01	0.7±0.05
20:4n-6	14.2±0.6	13.8±1.6
20:5n-3	0.1±0.05	0.3±0.03*
22:2n-6	0.2±0.05	0.2±0.06
22:5n-6	2.4±0.5	2.4±0.9
22:5n-3	0.3±0.04	0.8±0.1*
22:6n-3	1.6±0.5	2.5±1.7
Sum n-6 PUFA	32.9±1.6	31.5±3.2
Sum n-3 PUFA	2.0±0.5	3.6±1.7
n-6/n-3 ratio	17.6±3.9	9.8±2.9*

a data are mean ± SD of percent total FA.

b detected in one sample only.

c detected in two samples only.

*Significantly different (*P*<0.05) between Fat-1 and wild type mice.

**Table 2:** Mouse mammary gland triglyceride fatty acid composition.

Fatty acid	Wild type	Fat-1
14:0	1.3±0.07	1.4±0.2^a^
16:0	23.3±1.0	23.1±2.0
16:1n-7	5.3±0.7	4.5±0.7
18:0	2.4±0.2	2.7±0.3
18:1n-9	35.5±0.9	36.4±0.6
18:2n-6	29.6±0.9	30.3±1.3
18:3n-3	0.2±0.01	0.2±0.01
20:3n-6	0.2±0.01	0.2±0.02
20:4n-6	0.5±0.04	0.4±0.04
20:5n-3	0.02±0.004	0.03±0^b^
22:2n-6	0.05±0.02	0.03±0.01
22:5n-6	0.2±0.04	0.2±0.02
22:5n-3	0.02±0.005	0.3±0.004*
22:6n-3	0.05±0.01	0.07±0.008*
Sum n-6 PUFA	30.5±1.0	30.8±1.3
Sum n-3 PUFA	0.2±0.03	0.3±0.01*
n-6/n-3 ratio	128.2±15.8	107.9±5.5*

a data are mean ± SD of percent total FA.

b all samples had same value.

*Significantly different (*P*<0.05) between Fat-1 and wild type mice.

In liver tissues, in which the n-6/n-3 ratio in PL was 37% lower in Fat-1 than wt, DHA was the only detectable n-3 PUFA that was significantly elevated in Fat-1 compared to wt (Table 3). In liver TG, a 33% lower n-6/n-3 ratio (p = 0.06) was measured, mainly as a result of higher DPAn-3 in Fat-1 compared to wt tissue (Table 4).

**Table 3:** Mouse liver phospholipid fatty acid composition.

Fatty acid	Wild type	Fat-1
14∶0	0.1±0.01	0.1±0.02^a^
16∶0	19.5±1.8	19.3±0.9
16:1n-7	0.5±0.05	0.4±0.1
18∶0	19.6±1.1	19.9±1.1
18:1n-9	7.9±0.6	6.8±1.0
18:2n-6	14.1±1.1	14.5±0.5
18:3n-3	nd	nd
20:3n-6	1.4±0.2	1.1±0.2
20:4n-6	24.6±0.9	24.5±1.2
20:5n-3	nd	nd
22:2n-6	nd	nd
22:5n-6	4.5±0.9	3.5±0.7
22:5n-3	0.1^b^	0.2±0.01
22:6n-3	4.2±0.8	6.7±1.2*
Sum n-6 PUFA	44.6±1.3	43.6±1.4
Sum n-3 PUFA	4.3±0.7	6.9±1.3*
n-6/n-3 ratio	10.6±1.9	6.7±1.2*

a data are mean ± SD of percent total FA.

b one sample only.

nd none detected.

*Significantly different (*P*<0.05) between Fat-1 and wild type mice.

**Table 4:** Mouse liver triglyceride fatty acid composition.

Fatty acid	Wild type	Fat-1
14∶0	0.7±0.5	0.6±0.3^a^
16∶0	27.0±1.5	29.1±2.3
16:1n-7	3.1±1.6	1.7±1.0
18∶0	2.0±0.4	2.5±0.4
18:1n-9	37.6±2.9	34.3±1.1*
18:2n-6	22.0±2.6	24.0±2.9
18:3n-3	0.08±0.01	0.08±0.03
20:3n-6	0.6±0.2	0.7±0.2
20:4n-6	1.5±0.8	1.4±0.9
20:5n-3	nd	nd
22:2n-6	nd	nd
22:5n-6	0.5±0.2	0.8±0.3
22:5n-3	0.02±0.01	0.07±0.02*
22:6n-3	0.1±0.05	0.2±0.04*
Sum n-6 PUFA	24.6±3.4	27.4±2.3
Sum n-3 PUFA	0.16±0.06	0.27±0.08*
n-6/n-3 ratio	163±48	109±29

a data are mean ± SD of percent total FA.

nd none detected.

*Significantly different (*P*<0.05) between Fat-1 and wild type mice.

#### SDC-1 mRNA and protein is elevated in Fat-1 compared to wt mice

**Figure 2:**
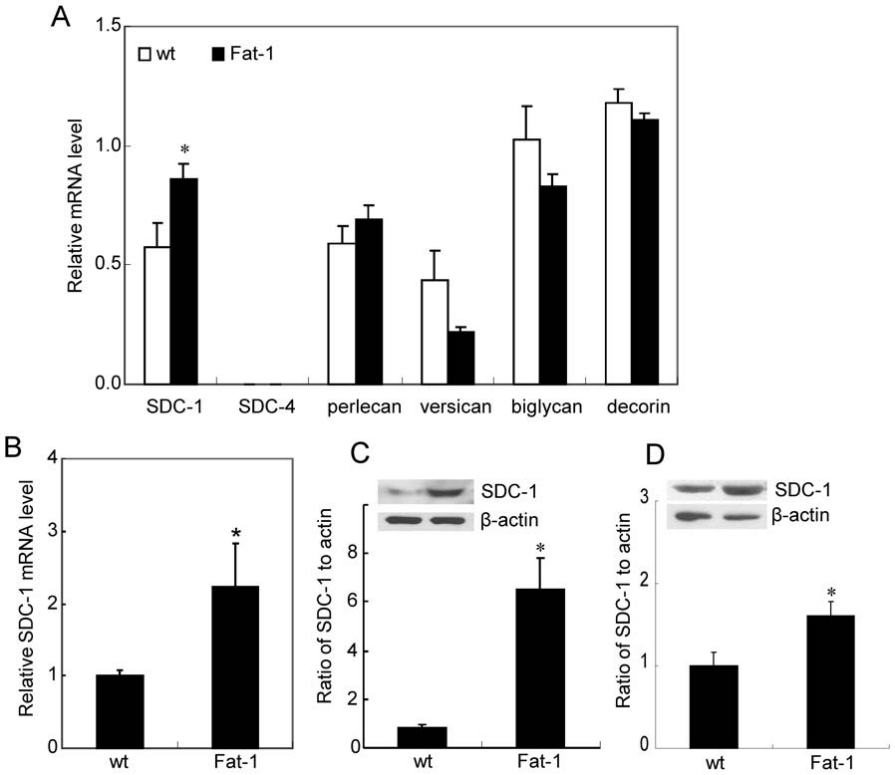
Expression of proteoglycans in tissues of Fat-1 and wild type (wt) mice. **A**. transcript levels of proteoglycans in mammary gland. **B**, transcript levels of SDC-1 in liver. Total RNA was isolated from tissues and mRNAs were measured by real-time RT-PCR. Numbers represent the level of expression relative to that of a housekeeping gene, peptidyl prolyl isomerase B (PPIB) using triplicate samples of each animal (n = 5). The expression of SDC-4 relative to PPIB in mammary tissue was <0.001 and similar between Fat-1 and wt tissues. **C** and **D**. Western analysis using a SDC-1 core protein-specific antibody of heparinase-treated protein extracts of **C**, mammary tissues and **D**, liver. Bars are means ± SEM (n = 5) of SDC-1 protein adjusted to β actin. Insert shows representative samples of wt and Fat-1bands. * *P*<0.05 Fat-1 versus wt by Student's *t* test.

**Figure 3:**
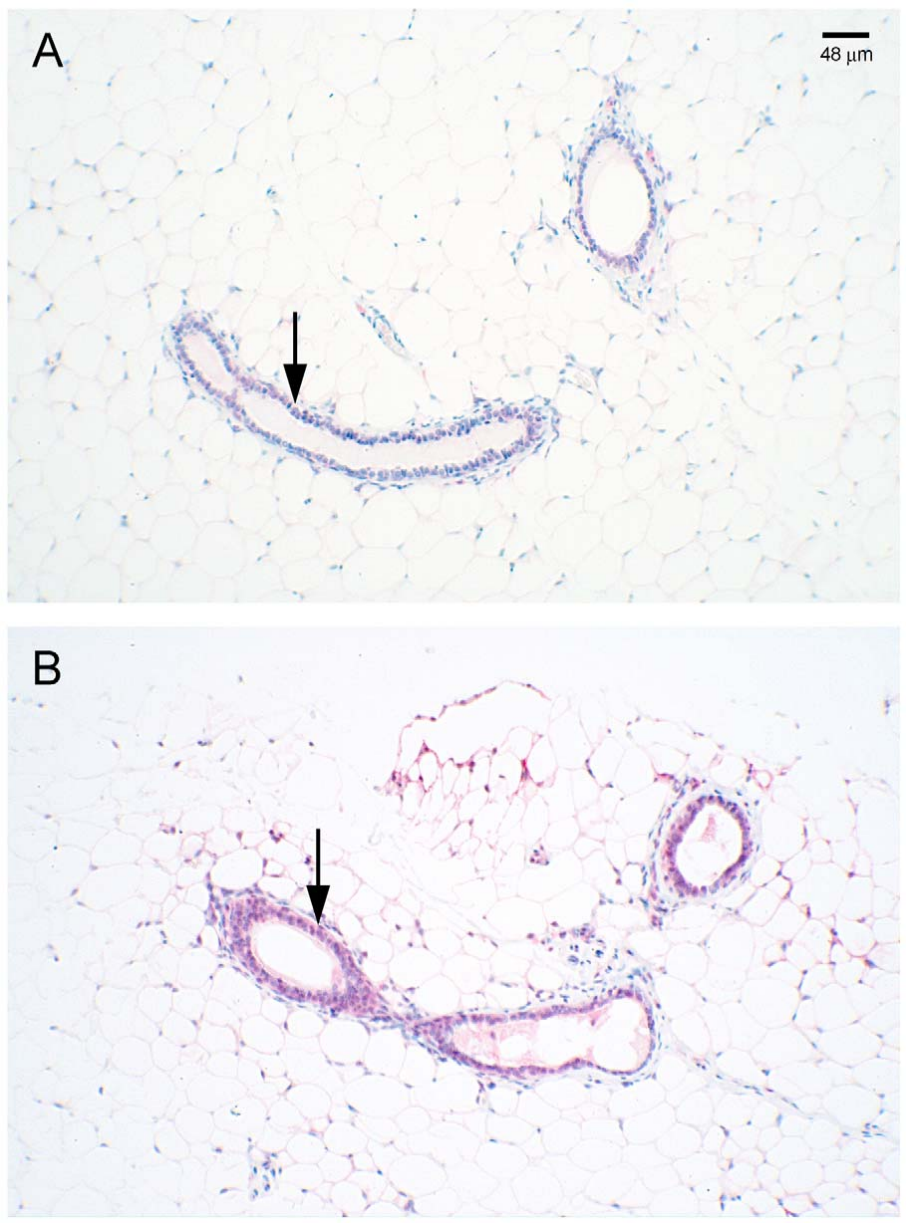
Immunostaining of SDC-1 in mammary glands of Fat-1 and wild type mice. Mammary glands from wt (**A**) and Fat-1 (**B**) mice were fixed, sectioned and immunostained with antibody H-174 raised against a recombinant protein corresponding to amino acids 82-256 of the human SDC-1 core protein. Arrows point to SDC-1 immunoreactive product localized in ductal epithelial cells.

SDC-1 is up-regulated when mammary cells are treated in vitro with n-3 PUFA [20], [22]. We investigated whether the modest increase in n-3 PUFA measured in the mammary tissues of the Fat-1 mice was sufficient to modify the expression of SDC-1 in vivo. As shown in Fig. 2A, mRNA for SDC-1 was 50% higher in mammary tissue of Fat-1 compared to wt mice. This effect was specific for SDC-1 since expression of the other proteoglycans, SDC-4, perlecan, decorin and biglycan were similar between Fat-1 and wt tissues (Fig. 2A). It is of interest to note that expression of the chondroitin sulfate proteoglycan, versican, which is generally thought to have a tumor-promoting effect, trended lower (p = 0.057) in the n-3 PUFA-enriched Fat-1 tissues. No previous studies have reported regulation of versican by n-3 PUFA. To determine whether SDC-1 expression was elevated in tissues other than mammary gland of Fat-1 mice, we examined liver where SDC-1 is known to function in the clearance of triglyceride-rich lipoprotein remnants [38]. As shown in Fig. 2B, SDC-1 mRNA was > two-fold higher in the livers of Fat-1 compared to wt animals. To confirm that the increased SDC-1 mRNA in Fat-1 tissues was translated into increased protein, Western analysis of tissue protein extracts demonstrated a six-fold higher SDC-1 protein level in mammary tissue (Fig. 2C) and a 50% higher SDC-1 protein in liver (Fig. 2D) of Fat-1 compared to wt mice. Finally, since whole mount preparations of mammary glands of Fat-1 mice were previously shown to display a more differentiated phenotype than wt animals [39], we examined SDC-1 distribution in the mammary tissues (Fig. 3). Immunostaining for SDC-1, localized primarily to epithelial components, was consistently more intense in Fat-1 than in wt tissues but no significant morphological differences were observed between the genotypes.

## Discussion

The conclusion from our studies is that endogenously synthesized n-3 PUFA in mouse mammary gland and liver are associated with an upregulation of SDC-1 expression in the absence of n-3 enriched LDL. This conclusion is drawn from the data showing that as a result of high n-6 PUFA dietary intake, the lipoproteins in both Fat-1 and wt mice are highly enriched in n-6 PUFA and lack detectable n-3 PUFA. Nevertheless, the mammary tissue and liver lipids of the Fat-1 group display an elevation in n-3 PUFA compared to wt as a result of the *fat-1* transgene. The increase in n-3 PUFA in these animals was very modest, but it was sufficient to be associated with increased expression of SDC-1 mRNA and protein. Although we have previously demonstrated increased expression of SDC-1 in prostate tissues of mice fed an n-3 PUFA enriched diet [40], the present study is the first time that this relationship has been investigated in mammary gland and liver in vivo and in a genetic model of n-3 PUFA regulation.

The Fat-1 mouse represents an excellent model for n-3 PUFA-gene regulation studies by eliminating variations in dietary fatty acid combinations. Kang et al [33] reported increased n-3 PUFA in all major tissues of this model including muscle, heart, brain, liver, kidney, lung, and spleen. Increases in n-3 PUFA reported in mammary gland by Ma et al [41] and in liver by Kang et al [33] were higher in Fat-1 mice than those of the present studies. This may be due to a dampening effect on the desaturase gene by the higher fat diet used in our studies. Further it was shown that in brain tissue, DHA levels achieved in this model were similar to those achieved by fish oil feeding [42]. An anti-tumor role for n-3 PUFA in Fat-1 mice was suggested by studies showing a reduced colitis-associated colon cancer that was associated with a decreased inflammatory response [43], [44]. In addition, the growth of implanted B16 human melanoma cells was markedly reduced in Fat-1 compared to wt mice [45]. Our previous studies have shown that introduction of the *fat-1* gene into a prostate specific Pten knock-out mouse model of prostate cancer reduced tumor growth similar to the effect of an n-3 PUFA diet [34]. Whether the increased n-3 PUFA would protect Fat-1 mice against breast cancer remains to be studied.

A few in vitro studies have examined biological and biochemical effects of expression of the *fat-1* transgene that result from the increased n-3 PUFA. In human prostate cancer cell lines transfected with the *fat-1* gene, proliferation was inhibited through a reduction in GSK-3β phosphorylation and a subsequent downregulation of β-catenin and cyclin D1[46]. In human lung cancer cells, the increased n-3 PUFA in *fat-1*-transfected cells resulted in reduced invasive properties due to down regulation of invasion-related genes, MMP-1 and integrin-α2 [47]. The studies described here present SDC-1 as a new candidate for regulation by the n-3 PUFA-enriched environment produced by the *fat-1* transgene. These data are consistent with previous studies showing vitro upregulation of SDC-1 by n-3 PUFA in human breast and prostate cancer cells as well as in vivo upregulation of SDC-1 in prostate tissue by an n-3 PUFA-enriched diet [20], [21], [22], [40]. We have further shown that SDC-1 plays a key role in the anti-tumor properties of n-3 PUFA through induction of apoptosis via inhibition of the PDK-1-Akt-Bad signaling pathway [22], [48].

In animals fed the n-6 PUFA diet, the similar increase in n-6/n-3 PUFA ratios of lipoproteins between the genotypes indicated that they directly reflected dietary PUFA intake with little influence of the *fat-1* transgene. Although, dietary effects on plasma lipids are strongest in the early postprandial period, mice will generally shift their tissue fatty acid compositions with time on diet to become more like the diet. Since the mice were fed the diet for several weeks, the differences in postprandial and fasting fatty acid compositions would be small. Further, the 4 h fasting period should mean that most of the postprandial chylomicrons have been cleared so that at 4 h, the effects of any difference in postprandial fatty acid compositions are minimal. Yet, in spite of similar lipoprotein fatty acids, the endogenously synthesized n-3 PUFA in the mammary and liver tissues was sufficient to increase SDC-1 expression. We have previously shown that DHA delivered by the LDL pathway in vitro is able to upregulate SDC-1 through activation of PPARγ [21], [22]. The results of the present study would be consistent with activation of PPARγ by endogenous n-3 PUFA but in addition raise the interesting possibility that in vivo, endogenously synthesized-PUFA and LDL-delivered PUFA may compartmentalize to impact different pathways of gene regulation. In addition, our study shows for the first time that n-3 PUFA upregulate syndecan-1 in the mouse liver, where this proteoglycan plays a primary role in triglyceride–rich lipoprotein clearance [38]. This suggests that through syndecan-1, n-3 PUFA may have beneficial effects on lipoprotein homeostasis relative to cardiovascular disease.

Although there are some conflicting reports [49], [50], evidence is strong for an anti-tumor role for n-3 PUFA, and human trials are showing promise that DHA combined with chemotherapy may improve outcome for breast cancer patients [51], [52]. However, not all studies have indicated a tumor inhibitory role for SDC-1 which appears to be pleiotropic depending on the tissue and cell of origin. In a variety of epithelial cancers, SDC-1 expression is reduced or totally lost with tumor progression and aggressive phenotype [53], [54], [55], [56], [57] and low SDC-1 expression was shown to be associated with poor prognostic outcome and shorter survival [54], [56], [58], [59], [60]. Other studies have shown that in breast, ovarian, and gastric cancer, the loss in cancer cell SDC-1 is accompanied by increased stromal SDC-1 expression and adverse clinical outcome [61], [62], [63], [64]. Clearly the SDC-1 content of a tissue depends on the combined output of heterogeneous cell types. In normal mammary tissue, such as in the Fat-1 and wt mice, adipocytes are a major cell type present (data not shown) whose contribution to the SDC-1 content is unknown.

Our data indicate upregulation of SDC-1 in the Fat-1 mouse at the level of both transcription and translation. An additional level of modification affecting the role of SDC-1 in any tissue relates to the composition and structure of its glycosaminoglycan (GAG) chains through which it interacts with multiple ligands to direct cellular responses. The GAG-bearing ectodomains of SDC-1 undergo physiological shedding as a result of proteolytic cleavage of their core proteins at a specific plasma membrane proximal site [65], [66]. Shedding results in a pool of soluble SDC-1 that competes for the same ligands as the membrane-bound co-receptor. The ectodomains specifically inhibit the in vitro growth of cancer cells [32], [67], [68]. However, the inhibitory activity can be reversed by heparanase that generates fragments of heparan sulfate that activate fibroblast growth factor-2 [69]. Heparanase is known to promote progression in a number of cancers [70] and its presence may be a major determinant on whether SDC-1 plays a tumor-promoting or tumor inhibitory role [71].

In summary, studies to date indicate a complicated relationship between SDC-1 and cancer. One direction that may help to unravel its role in cancer is to examine the regulation of SDC-1 by tumor suppressive or promoting agents. Our previous studies strongly supported an upregulation of SDC-1 by n-3 PUFA that resulted in reduced growth and apoptosis induction in breast cancer cells in vitro [21], [22]. The present study has confirmed in vivo elevation of SDC-1 in n-3 PUFA-enriched mammary tissues of the Fat-1 mouse.
